# Nodal asymmetry and hedgehog signaling during vertebrate left–right symmetry breaking

**DOI:** 10.3389/fcell.2022.957211

**Published:** 2022-09-12

**Authors:** Maria Isabella Negretti, Nina Böse, Natalia Petri, Stanislav Kremnyov, Nikoloz Tsikolia

**Affiliations:** ^1^ Anatomy and Embryology, University Medical Center Göttingen, Göttingen, Germany; ^2^ Department of Embryology, Faculty of Biology, Lomonosov Moscow State University, Moscow, Russia; ^3^ Koltzov Institute of Developmental Biology, Russian Academy of Sciences, Moscow, Russia

**Keywords:** left–right (LR) asymmetry, chick embryo, nodal, hedgehog signaling, heart looping, *Xenopus laevis* embryo, embryonic competence

## Abstract

Development of visceral left–right asymmetry in bilateria is based on initial symmetry breaking followed by subsequent asymmetric molecular patterning. An important step is the left-sided expression of transcription factor *pitx2* which is mediated by asymmetric expression of the *nodal* morphogen in the left lateral plate mesoderm of vertebrates. Processes leading to emergence of the asymmetric *nodal* domain differ depending on the mode of symmetry breaking. In *Xenopus laevis* and mouse embryos, the leftward fluid flow on the ventral surface of the left–right organizer leads through intermediate steps to enhanced activity of the nodal protein on the left side of the organizer and subsequent asymmetric *nodal* induction in the lateral plate mesoderm. In the chick embryo, asymmetric morphogenesis of axial organs leads to paraxial *nodal* asymmetry during the late gastrulation stage. Although it was shown that hedgehog signaling is required for initiation of the *nodal* expression, the mechanism of its asymmetry remains to be clarified. In this study, we established the activation of hedgehog signaling in early chick embryos to further study its role in the initiation of asymmetric *nodal* expression. Our data reveal that hedgehog signaling is sufficient to induce the *nodal* expression in competent domains of the chick embryo, while treatment of *Xenopus* embryos led to moderate *nodal* inhibition. We discuss the role of symmetry breaking and competence in the initiation of asymmetric gene expression.

## Introduction

The position and shape of visceral organs of adult vertebrates do not display mirror symmetry deviating from the midline (Matsui and Bessho, 2012; Hamada and Tam, 2020). This left–right asymmetry is a fundamental morphological feature of all studied vertebrates and of many studied invertebrates (Tisler et al., 2016; Datar et al., 2017; Blum and Ott, 2018; Lebreton et al., 2018). The asymmetry displays a definite direction typical for the organism in the vast majority of cases (Afzelius and Stenram, 2006). This “normal” position, also called *situs solitus*, is based on asymmetric morphogenetic processes which are believed to be preceded by asymmetric gene expression. The key asymmetric “highway” is created by the left-sided expression of the TGF-beta ligand *nodal* and its downstream counterpart transcription factor *pitx2* in the lateral plate mesoderm (Levin et al., 1995; Logan et al., 1998; Rebagliati et al., 1998; Campione et al., 1999; Patel et al., 1999; Blum et al., 2009; Grande and Patel, 2009). The asymmetry of the *nodal* expression is at the same time an early marker of successful symmetry breaking which is achieved in vertebrates by at least two different mechanisms. The so-called leftward flow is based on the rotation of long monocilia on the ventral surface of the epithelial progenitors of the axial mesoderm and operates in most model organisms such as *X. laevis*, *Danio rerio*, and *Mus musculus* (Essner et al., 2005; Nonaka et al., 2005; Schweickert et al., 2007), where it is suggested to be a symmetry breaking mechanism; also, earlier relevant asymmetries have been reported and suggested to break the symmetry, particularly in *X. laevis* (Lobikin et al., 2012; Vandenberg et al., 2013; Onjiko et al., 2016). The direction of the leftward flow is detected by sensory monocilia on the so-called somitic floor plate. This leads through intermediate steps to the left-sided activation of the paraxial secreted nodal protein, which in turn is responsible for its subsequent expression in the left lateral plate mesoderm (Schweickert et al., 2010; Maerker et al., 2021). However, the first signs of the molecular asymmetry in the chick and some mammals are seen already in the paraxial mesoderm in the form of the left-sided *nodal* domain (Tsikolia et al., 2012; Schroder et al., 2016), indicating divergence of symmetry-breaking mechanisms in vertebrates (Kajikawa et al., 2020). This suggestion is supported by both the absence of morphological structures enabling flow (Manner, 2001; Tsikolia et al., 2012; Stephen et al., 2014; Pieper et al., 2020) and the evolutionary loss of genes involved in the nodal activating network operating downstream of the leftward flow (Szenker-Ravi et al., 2022). Asymmetric *nodal* expression in the chick is preceded by a leftward node rotation at stage 4 (Cui et al., 2009; Gros et al., 2009), which is followed by asymmetric positioning of the axial mesoderm and the floor plate anlagen (Otto et al., 2014). As a result, the posterior part of the floor plate lies to the left of the notochord, whereby the expression of the *sonic hedgehog* (*shh*) morphogen in the floor plate is located directly above the prospective *nodal* domain in the left paraxial mesoderm, hence indicating a possible functional relationship. Sonic hedgehog is an early marker expressed in the organizer region and emerged in midline structures in various model organisms (Echelard et al., 1993; Roelink et al., 1994; Kremnyov et al., 2018) and is involved as a secreted morphogen in molecular patterning at different sites including neural tube and somitogenesis (Johnson et al., 1994; Ribes et al., 2010; Cohen et al., 2015). The activity of hedgehog signaling is required for initiation of the paraxial *nodal* expression in the chick as shown both by local anti-hedgehog antibody application (Pagan-Westphal and Tabin, 1998) and by global inhibition of hedgehog signaling by cyclopamine (Otto et al., 2014). Moreover, the *nodal* domain overlaps with the hedgehog receptor *patched1* expression, which serves as an indirect reporter of hedgehog activity. Local implantation of *shh* expressing cells or of beads soaked with *shh* protein right to the node activates expression of *nodal* or its downstream effector *pitx2* in the lateral plate mesoderm (LPM) at stage 9, indicating that *shh* is sufficient for *nodal* induction (Levin et al., 1995; Logan et al., 1998). However, the effect of ectopic *shh* on early paraxial *nodal* expression has not been studied yet. Moreover, hedgehog signaling undergoes complex regulation, and the presence of *shh* does not necessarily lead to an activated pathway. For example, the absence of *shh* and its downstream effector smoothened cause opposite effects in the mouse embryo (Tsukui et al., 1999; Tsiairis and McMahon, 2009), and phenotypic effects of knocking out of different hedgehog components on neural tube development are ambiguous (Motoyama et al., 2003; Iulianella et al., 2018). Hence, we aimed to test the effect of downstream signaling activation on the early left–right patterning and asymmetry by non-local activation of hedgehog signaling downstream of *shh* using the smoothened activator SAG. Furthermore, the global activator treatment enables addressing the competence of tissue. Our clear-cut results in the chick inspired us to examine the influence of hedgehog activation on *nodal1* expression in the lateral plate mesoderm in *X. laevis* since the functional studies of hedgehog signaling indicated its non-conserved role in the left–right patterning (Tsiairis and McMahon, 2009; Zhu et al., 2020).

## Materials and methods

### Embryos

Fertilized chick eggs were incubated at 38°C under humidified conditions until reaching the desired stage (Hamburger and Hamilton, 1951; Pieper et al., 2020). For *in vitro* culture, embryos were processed as previously described (Sydow et al., 2017). After cultivation, blastoderms were prefixed in a fixative, excised, transferred into a petri dish, rinsed in Locke’s solution, and fixed in 4% PFA in phosphate-buffered saline (PBS) for 1 h.

*X. laevis* embryos were obtained by hormone-induced egg laying and *in vitro* fertilization using standard methods (Kay and Peng, 1992), de-jellied in 2% L-cysteine solution, pH 8, and then cultured in 0.1X MMR at 14–18°C. The embryos were staged according to the tables of normal development (Nieuwkoop and Faber, 1994). Before fixation in 4% PFA in phosphate-buffered saline (PBS), the embryos were liberated from the vitelline membrane with forceps. For further details, see Supporting Methods.

### SAG treatment

*In vivo* activation of hedgehog signaling was performed with smoothened activator SAG (Bragina et al., 2010; Lewis and Krieg, 2014; Meinhardt et al., 2014; Todd and Fischer, 2015). SAG (Sigma-Aldrich, Taufkirchen) was dissolved in Millipore water to 10 mM (stock solution). The stock solution was diluted in PBS (for chicken) or 0.1X MMR (frog) to a final concentration of 250 and 100 µM, respectively, which were determined in pretests with different readouts. There, we also found that SAG retains its activity for 6 months if stored at −20°C. Control embryos were treated with 1% (*Xenopus*) or 2.5% (for chicken) Millipore water diluted in 0.1X MMR or PBS, respectively. Treatment of chick embryo was performed by application of 10 μl of the corresponding solution twice within 2 h to the ventral side of the embryos cultivated in a modified new culture as previously reported (Otto et al., 2014; Sydow et al., 2017).

### *In situ* hybridization and cloning

*In situ* hybridization was performed according to previously published protocols (Harland, 1991; Weisheit et al., 2002). Digoxigenin-labeled mRNA probes for *nodal* and *pitx2* were produced using the previously published plasmid DNAs (Levin et al., 1995; Logan et al., 1998). For further details, see Supplementary Methods.

## Results

**FIGURE 1:**
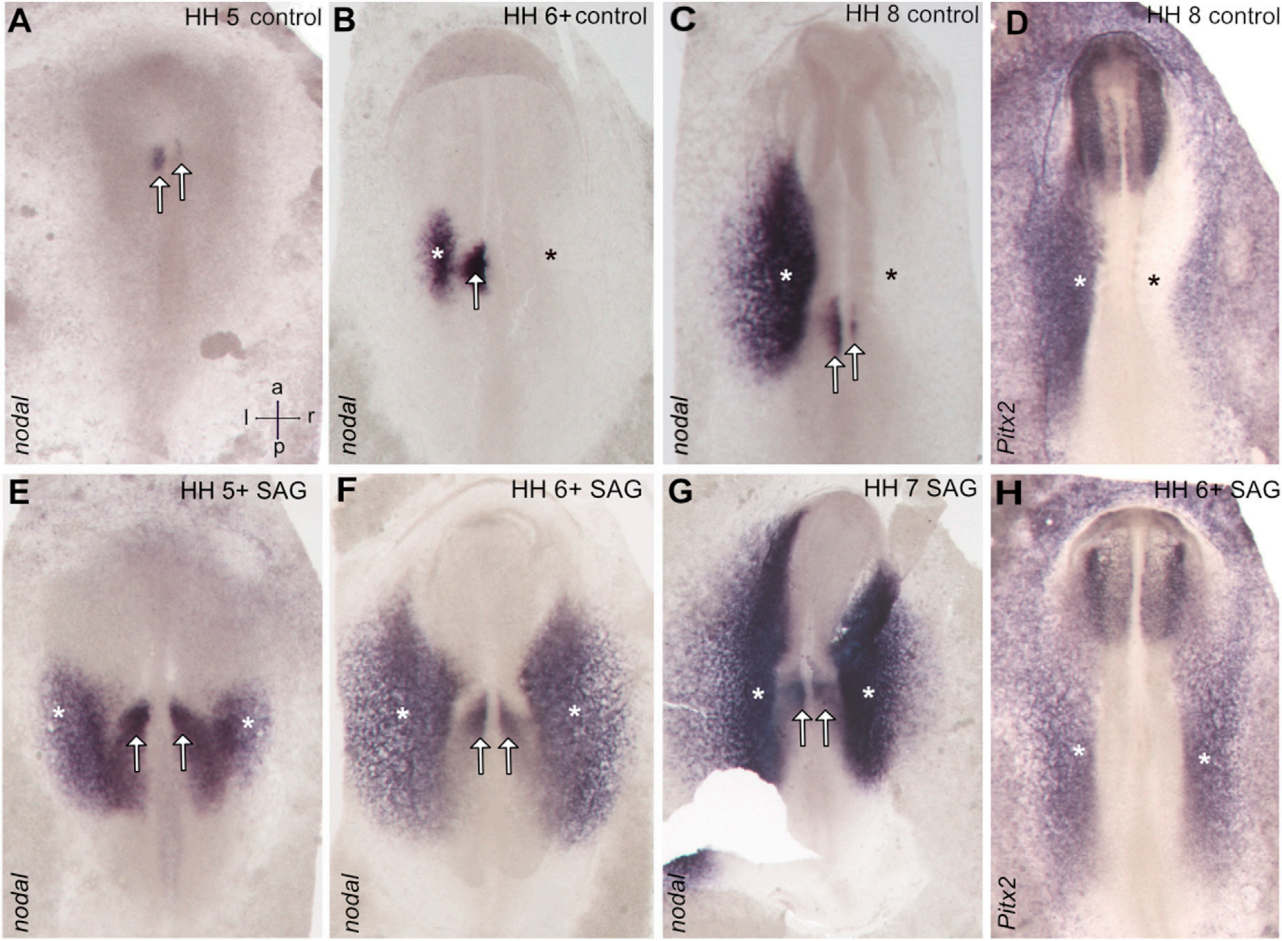
Symmetrical expression of markers of left–right patterning between stages 5 and 7 in the chick embryos treated with the activator of hedgehog signaling SAG. In control embryos, *nodal* expression is confined to the left-sided domains close to the posterior midline (paraxial domain) and in the area of the lateral plate mesoderm **(A–C),** while treated embryos reveal bilateral expression. **(E–G)** Similarly, *pitx2* reveals in treated embryos bilateral expression in the lateral plate mesoderm. **(H)**, while control embryos reveal left-sided expression in this domain **(D)**. Asterisks indicate the position of the lateral plate mesoderm, arrows the paraxial domain, and intersecting arrows anatomical axes.

**FIGURE 2:**
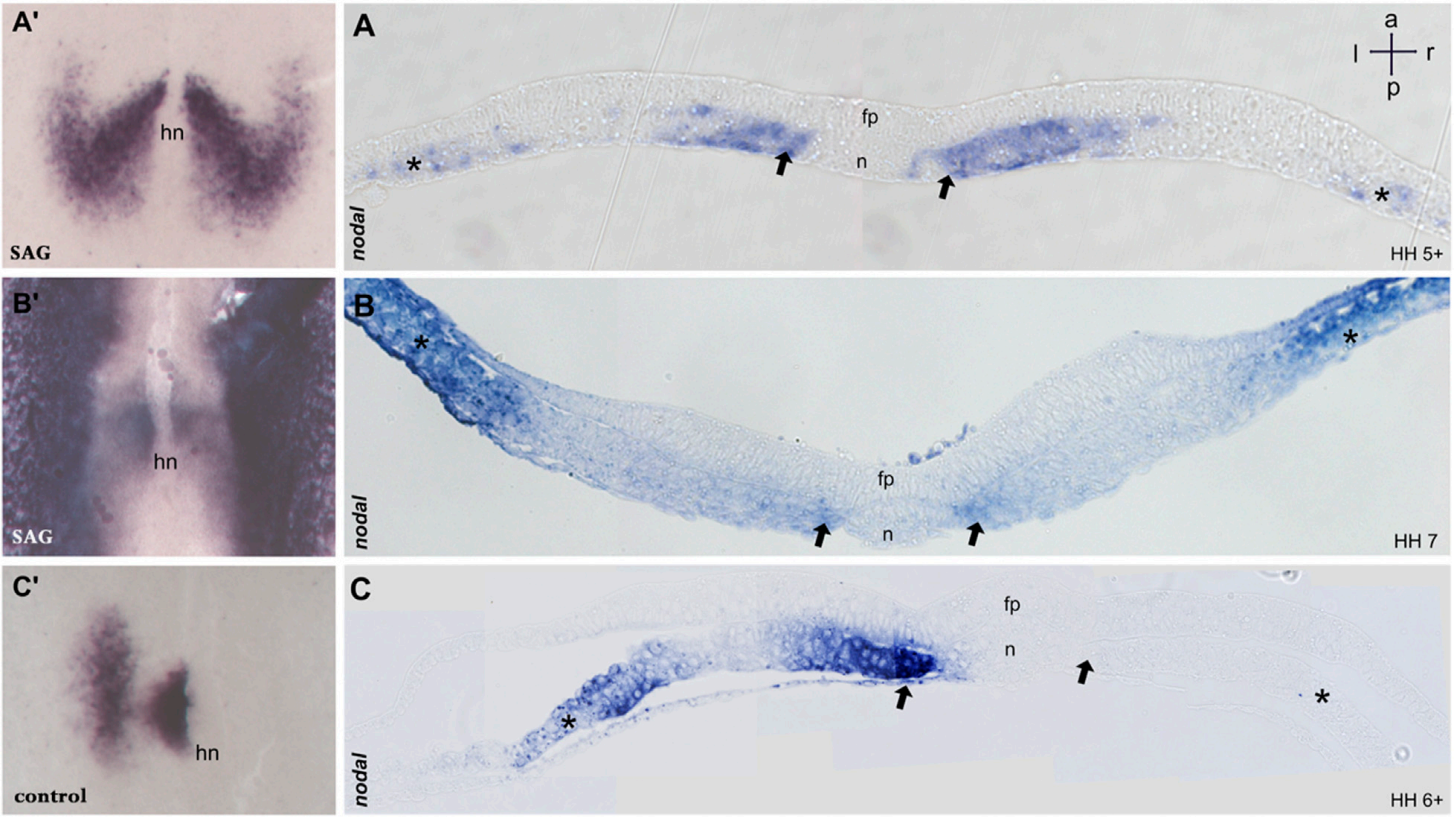
Sections of embryos treated with SAG. **(A–C)** 5 μm transversal sections of plastic (Technovit) embedded embryos at the level indicated in **A′–C′,** respectively. At stage 5, *nodal* expression is confined to the paraxial mesoderm and to mesodermal cells located laterally **(A,A′)**. Stage 6 reveals four distinct domains confined to the paraxial mesoderm and lateral plate mesoderm on both sides **(B,B′)**. Control embryos at stage 6 reveal expression in the left paraxial and lateral plate mesoderm **(C,C′)**. n, notochord; fp, floor plate; hn, Hensen’s node; asterisk, lateral plate mesoderm; arrow, paraxial mesoderm. Intersecting arrows indicate anatomical axes.

Stage 4 chick embryos were treated with control solution or smoothened agonist SAG and cultivated until stages 5–7. The control embryos show asymmetric *nodal* expression which at stage 5 reveals a strong narrow domain left to the notochord located in the paraxial mesoderm (Figure 1A), where advanced stage 6 expression is confined to both the left paraxial mesoderm and the lateral plate mesoderm (LPM), while these domains come close at the posterior level (Figures 1B, 2C, 2C'). From stage 7, the domain in the LPM extends and elongates especially toward the anterior pole (Figure 1C). The asymmetry of *nodal* expression is seen at all stages and is particularly strong in the LPM domain where it was never seen on the right side.

Treated embryos develop without delay and form the notochord, head fold, and somites, while the overall phenotype was slightly widened. *In situ* hybridization reveals a strong and bilateral nodal expression. From advanced stage 5, the paraxial expression extends in the posterior and the lateral direction (Figures 1E, 2A′), forming a V-shaped domain. Sections reveal expression in emerging paraxial and lateral plate mesoderm (Figure 2A). At stages 6 and 7, *nodal* expression is confined to distinct elongated paraxial domains lateral to the notochord and to broad bilateral domains in the LPM (Figures 1F,G, 2B′,B), particularly at stage 6. Generally, *in situ* hybridization revealed strong symmetrization of the *nodal* domain (Supplementary Table S1A). We also analyzed the expression of *nodal* downstream effector *pitx2*. In control embryos, *pitx2* expression at stages 7 and 8 reveals expression in extraembryonic tissue as well as in the left lateral plate mesoderm (Figure 1D). In treated embryos (Supplementary Table S1B), expression is confined particularly to the right lateral plate mesoderm (Figure 1H).

**FIGURE 3:**
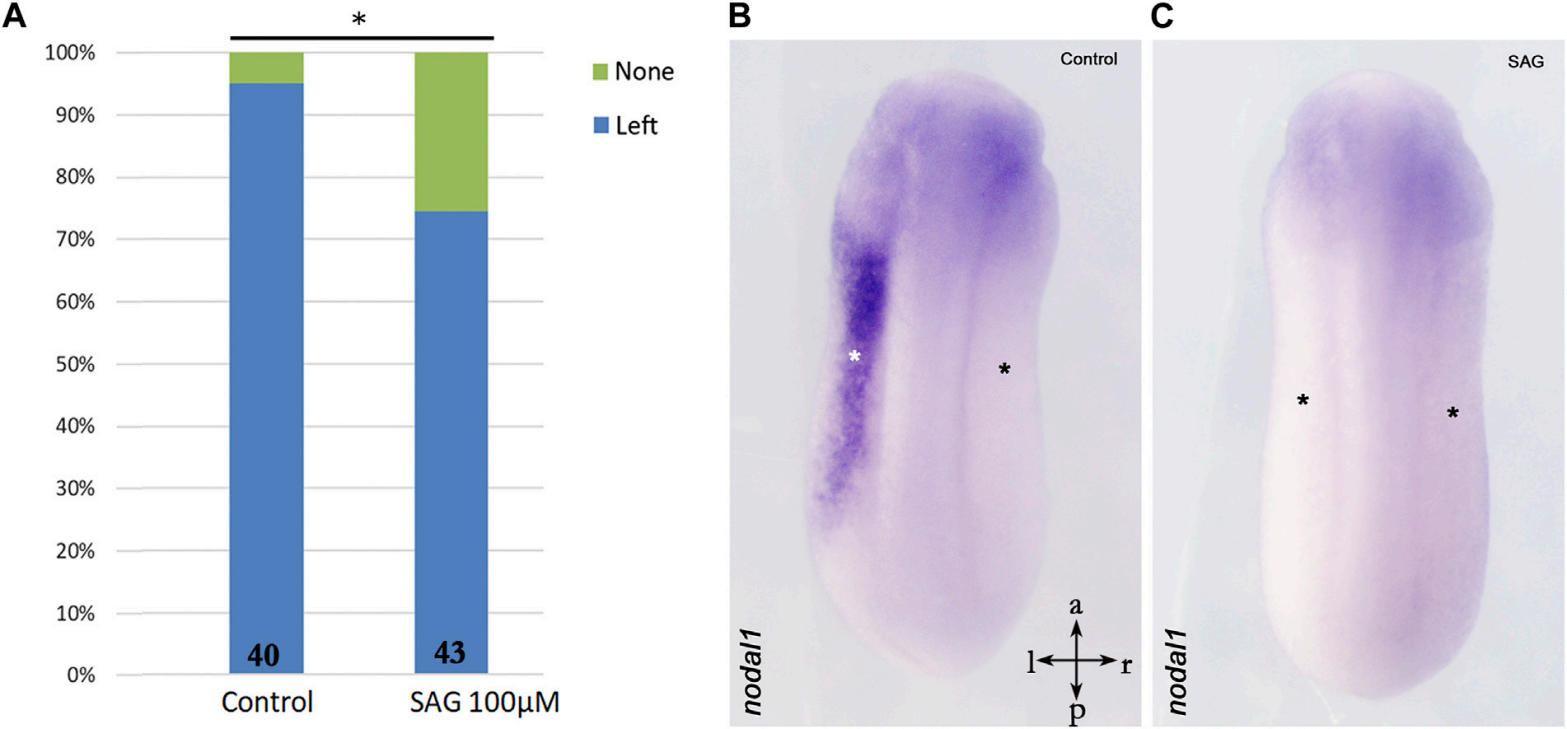
Inhibition of *nodal1* expression in the lateral plate mesoderm of *X. laevis* embryos after treatment with an activator of hedgehog signaling. **(A)** Treatment with SAG leads to inhibition of *nodal1* expression as compared to control embryos. **(B)** Example of left-sided *nodal1* expression; **(C)** example of suppressed *nodal1* expression. Intersecting arrows indicate anatomical axes. Numbers at the base of columns represent a number of analyzed embryos. *p < 0.05 (0.023) as compared to control and two-proportions z-test.

Furthermore, we treated stage 9 *X. laevis* embryos with SAG and analyzed *nodal1* expression in the LPM at stage 24. Surprisingly, expression analysis indicates significant inhibition of *nodal1* in the lateral plate mesoderm as compared to the control embryos (Figure 3).

**FIGURE 4:**
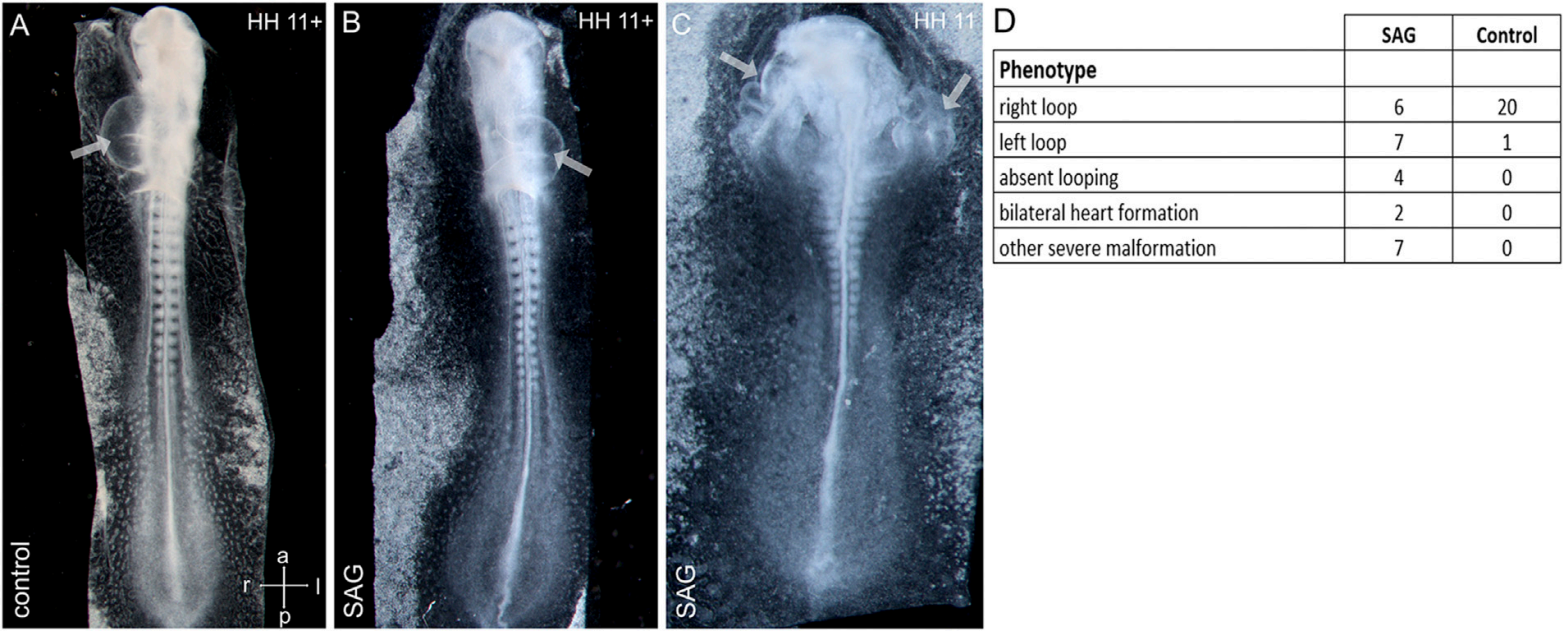
Abnormal cardiac development after treatment with an activator of hedgehog signaling SAG. **(A–C)**: examples of right-sided **(A)**, left-sided looping **(B),** and bilateral heart development **(C)** after SAG treatment. **(D)** Distribution of cardiac phenotypes in control and treated embryos. Arrows indicate heart tubes. Intersecting arrows indicate anatomical axes.

Asymmetrical cardiac looping is the first morphological sign of left–right asymmetry shared by all known model vertebrates (Manner, 2009; Lombardo et al., 2019). To study the influence of SAG on early morphological asymmetry, we treated chick embryos at stage 4 and let the embryos develop until stages 11–12. Treatment led to both randomizations of the cardiac looping and absent looping with a widened heart and severe deformities of the cardiac formation related to abnormal development of the intestinal portal, which included duplications of the heart tube (Figure 4).

## Discussion

The activity of sonic hedgehog has been proposed to be upstream of the asymmetric *nodal* expression in the chick as supported both by the spatial relationship between *shh* and *nodal* domains as well as by functional studies (cf. Supplementary Table S2) that indicate the involvement of *shh* or hedgehog signaling for *nodal* activation in the paraxial and LPM-domains (Levin et al., 1995; Pagan-Westphal and Tabin, 1998; Otto et al., 2014). The aim of our study was to test the spatio-temporal effects of global activation of hedgehog signaling in the framework of the early left–right patterning. Treatment with smoothened agonist SAG which activates hedgehog signaling caused additional *nodal* expression on the right side, hence mirroring the left-sided domain, elevating expression intensity and leading to decoupling of initiation of *nodal* in LPM from the beginning of somitogenesis. A medial sector of widened *nodal* expression at stage 5 after SAG treatment overlaps with the expression of the somitic mesoderm marker *paraxis* at stage 5 (not shown), indicating a *nodal* induction in the prospective somitic mesoderm. Right-sided *nodal* expression correlates with the right-sided expression of *pitx2* in LPM. Furthermore, the SAG treatment interferes with cardiac development. While inhibition and local activation of hedgehog signaling lead to randomization of cardiac looping (Levin et al., 1997; Logan et al., 1998; Otto et al., 2014), the global activation leads to both randomization and severe deformations associated with disturbed closure of the intestinal portal. The latter may be explained by the involvement of *shh*, which is expressed in the endoderm (Narita et al., 1998), in the morphogenesis of the intestinal portal.

Our results argue in favor of a crucial role of competence in the *nodal* induction during chick left–right patterning: despite the treatment of the whole embryos with SAG, the ectopic *nodal* expression was confined to the paraxial and the lateral plate mesoderm. As suggested previously, the left paraxial *nodal* domain is most likely induced by *shh* from the floor plate above (Supplementary Figure S1). This induction, however, is specifically constrained: particularly, SAG-treated embryos reveal at the right side a competent domain mirroring the left domain. The concept of embryonic competence describes the ability or predisposition of the embryonic tissue to produce a specific response to a certain signal (Waddington, 1932; Waddington, 1936; Gurdon, 1987). The theoretical significance of competence lies in the fact that it points to the not yet discovered layer of interactions (Tsikolia, 2006). It may not only rely on the pattern of cellular receptivity on different levels but *sensu lato* also on specific transport and degradation of signaling molecules, enabling a local response to the global cue. The mechanism of predisposition of a certain mesodermal domain at the right side to respond to hedgehog activity with the *nodal* expression remains to be clarified: reported expression of the hedgehog pathway components *gli* or *smoothened* only partially overlaps with the prospective *nodal* expression domain. Further studies should clarify the basis of competence as well as the question of whether the lateral plate mesoderm in the chick is induced solely by a signal from the paraxial domain or possesses the ability to express *nodal* autonomously.

SAG has been used as a hedgehog activator in different models (Bragina et al., 2010; Meinhardt et al., 2014; Todd and Fischer, 2015) and was suggested to be highly specific (Lewis and Krieg, 2014). Although the question concerning the specificity of reported SAG effect on *nodal* expression cannot be definitively resolved here, the complementary effects of hedgehog suppression that led to efficient *nodal* inhibition and of proposed hedgehog activation by SAG which induced *nodal*, strongly support the specific involvement of hedgehog activation in the reported effect. Furthermore, the toxicity of SAG treatment until the organogenesis stages was low.

Activation of hedgehog signaling in our study led to different effects in chick and *Xenopus* embryos. This functional divergence is in line with previously published data from other organisms. In the sea urchin, the inhibition of hedgehog signaling by cyclopamine was shown to suppress both asymmetric *nodal* and *pitx2* expression (Warner et al., 2016). In amphioxus, the downregulation of hedgehog signaling causes bilateral *nodal* expression (Hu et al., 2017), while the upregulation leads to bilateral expression of *nodal* antagonist *DAND5* (Zhu et al., 2020). Interestingly, the asymmetric hedgehog activity in amphioxus was proposed to be caused by cilia-driven transport of hedgehog protein. Mouse embryos lacking smoothened or both shh and its relative Indian hedgehog (Ihh) do not express *nodal* in the lateral plate mesoderm (Tsiairis and McMahon, 2009). However, other studies reported bilateral symmetrical *nodal* expression in mouse embryos lacking *shh* alone (Tsukui et al., 1999), indicating a possible feedback loop between *shh* and Ihh in the mouse. Moreover, mouse embryos lacking both smoothened and hedgehog repressor Gli3 display a correct left-sided *pitx2* expression, indicating a functional redundancy (Tsiairis and McMahon, 2009)*.* Hence, detailed investigation of the exact mode of hedgehog activity in organisms with the leftward flow like *Xenopus*, mouse, or *D. rerio* can uncover unexpected levels of regulation.

To sum up, combined empirical evidence (cf. Supplementary Table S2) reveals that in the chick embryos, the hedgehog signaling is both necessary (Pagan-Westphal and Tabin, 1998; Otto et al., 2014) and sufficient (Levin et al., 1995 and this work) for initiation of *nodal* expression at stage 5 in the competent paraxial domain. We suggest that this interaction is local and the asymmetry of *nodal* is achieved by the left-sided position of the posterior floor plate (Supplementary Figure S1).

Furthermore, our data support the evolutionary divergence of *shh*-*nodal* interaction (Supplementary Figure S2) and provide an efficient model of stable symmetrization of *nodal* gene expression in the chick embryo.

## Data Availability

The original contributions presented in the study are included in the article/Supplementary Material; further enquiries can be directed to the corresponding author.

## Supplementary material

SUPPLEMENTARY TABLE S1 Left–right patterning in SAG-treated embryos and control: nodal and **(A)** pitx2 **(B)** expression.

SUPPLEMENTARY TABLE S2 Hedgehog signaling in the chick left–right patterning: compilation of previous results concerning the role of hedgehog signaling in left–right patterning in comparison to the present report.

SUPPLEMENTARY FIGURE S1 Proposed mechanism of molecular left‐right patterning as a consequence of asymmetric node morphogenesis in the chick. **(A)**: Schematic dorsal view of chick embryo at early stage 5 after node rotation. **(B,C)**: Transversal sections at levels shown in **(A)**. The notochord is marked in red, the floor plate is labeled in turquoise and blue indicates the paraxial nodal domain. Dashed arrows in **(A)** indicate a shift of the node during notochord elongation and concomitant streak regression, black arrow in **(C)** indicates the proposed induction of the paraxial nodal expression by shh from the notochord, green arrow in **(C)** indicates the proposed subsequent induction of nodal expression in the prospective lateral plate mesoderm (lpm) by nodal ligand from the paraxial domain. A is modified version of Figure 7B published in (Kremnyov et al., 2018).

SUPPLEMENTARY FIGURE S2 Hedgehog signaling and left‐right patterning in Deuterostomia. The red font is used for observed stimulating effect which is subdivided into “sufficient” and “required” (necessary) for nodal expression while the blue font is used for reported inhibition of nodal domain.

SUPPLEMENTARY DATA SHEET 1 Supplementary Methods

## References

[B1] Afzelius B. A. Stenram U. (2006). Prevalence and genetics of immotile-cilia syndrome and left-handedness. Int. J. Dev. Biol. 50 (6), 571–573. 10.1387/ijdb.052132ba 16741872

[B2] Blum M. Beyer T. Weber T. Vick P. Andre P. Bitzer E. (2009). Xenopus, an ideal model system to study vertebrate left-right asymmetry. Dev. Dyn. 238 (6), 1215–1225. 10.1002/dvdy.21855 19208433

[B3] Blum M. Ott T. (2018). Animal left-right asymmetry. Curr. Biol. 28 (7), R301–R304. 10.1016/j.cub.2018.02.073 29614284

[B4] Bragina O. Sergejeva S. Serg M. Zarkovsky T. Maloverjan A. Kogerman P. (2010). Smoothened agonist augments proliferation and survival of neural cells. Neurosci. Lett. 482 (2), 81–85. 10.1016/j.neulet.2010.06.068 20600593

[B5] Campione M. Steinbeisser H. Schweickert A. Deissler K. van BebberF. Lowe L. A. (1999). The homeobox gene Pitx2: Mediator of asymmetric left-right signaling in vertebrate heart and gut looping. Development 126 (6), 1225–1234. 10.1242/dev.126.6.1225 10021341

[B6] Cohen M. Kicheva A. Ribeiro A. Blassberg R. Page K. M. Barnes C. P. (2015). Ptch1 and Gli regulate Shh signalling dynamics via multiple mechanisms. Nat. Commun. 6, 6709. 10.1038/ncomms7709 25833741 PMC4396374

[B7] Cui C. Little C. D. Rongish B. J. (2009). Rotation of organizer tissue contributes to left-right asymmetry. Anat. Rec. 292 (4), 557–561. 10.1002/ar.20872 PMC2714534 19301278

[B8] Datar A. Nandi S. Julicher F. Grill S. (2017). Left-right symmetry breaking during early development of *C. elegans* . Biophysical J. 112 (3), 434a. 10.1016/j.bpj.2016.11.2319

[B9] Echelard Y. Epstein D. J. St-Jacques B. Shen L. Mohler J. McMahon J. A. (1993). Sonic hedgehog, a member of a family of putative signaling molecules, is implicated in the regulation of CNS polarity. Cell 75 (7), 1417–1430. 10.1016/0092-8674(93)90627-3 7916661

[B10] Essner J. J. Amack J. D. Nyholm M. K. Harris E. B. Yost H. J. (2005). Kupffer's vesicle is a ciliated organ of asymmetry in the zebrafish embryo that initiates left-right development of the brain, heart and gut. Development 132 (6), 1247–1260. 10.1242/dev.01663 15716348

[B11] Grande C. Patel N. H. (2009). Nodal signalling is involved in left-right asymmetry in snails. Nature 457 (7232), 1007–1011. 10.1038/nature07603 19098895 PMC2661027

[B12] Gros J. Feistel K. Viebahn C. Blum M. Tabin C. J. (2009). Cell movements at Hensen's node establish left/right asymmetric gene expression in the chick. Science 324 (5929), 941–944. 10.1126/science.1172478 19359542 PMC2993078

[B13] Gurdon J. B. (1987). Embryonic induction--molecular prospects. Development 99 (3), 285–306. 10.1242/dev.99.3.285 3308408

[B14] Hamada H. Tam P. (2020). Diversity of left-right symmetry breaking strategy in animals. F1000Res 9, F1000. 10.12688/f1000research.21670.1 PMC7043131 32148774

[B15] Hamburger V. Hamilton H. L. (1951). A series of normal stages in the development of the chick embryo. J. Morphol. 88, 49–92. 10.1002/jmor.1050880104 24539719

[B16] Harland R. M. (1991). *In situ* hybridization: An improved whole-mount method for Xenopus embryos. Methods Cell Biol. 36, 685–695. 10.1016/s0091-679x(08)60307-6 1811161

[B17] Hu G. Li G. Wang H. Wang Y. (2017). Hedgehog participates in the establishment of left-right asymmetry during amphioxus development by controlling Cerberus expression. Development 144 (24), 4694–4703. 10.1242/dev.157172 29122841

[B18] Iulianella A. Sakai D. Kurosaka H. Trainor P. A. (2018). Ventral neural patterning in the absence of a Shh activity gradient from the floorplate. Dev. Dyn. 247 (1), 170–184. 10.1002/dvdy.24590 28891097 PMC5739940

[B19] Johnson R. L. LaufEr E. Riddle R. D. Tabin C. (1994). Ectopic expression of Sonic hedgehog alters dorsal-ventral patterning of somites. Cell 79 (7), 1165–1173. 10.1016/0092-8674(94)90008-6 8001152

[B20] Kajikawa E. Horo U. Ide T. Mizuno K. Minegishi K. Hara Y. (2020). Nodal paralogues underlie distinct mechanisms for visceral left-right asymmetry in reptiles and mammals. Nat. Ecol. Evol. 4 (2), 261–269. 10.1038/s41559-019-1072-2 31907383

[B21] Kay B. K. Peng H. B. (1992). *Xenopus laevis*: Practical uses in cell and molecular biology. Academic Press. 1811160

[B22] Kremnyov S. Henningfeld K. Viebahn C. Tsikolia N. (2018). Divergent axial morphogenesis and early shh expression in vertebrate prospective floor plate. Evodevo 9, 4. 10.1186/s13227-017-0090-x 29423139 PMC5791209

[B23] Lebreton G. Geminard C. LaprazF. PyrpaSSopouloS S. Cerezo D. SPeder P. (2018). Molecular to organismal chirality is induced by the conserved myosin 1D. Science 362 (6417), 949–952. 10.1126/science.aat8642 30467170 PMC6698710

[B24] Levin M. Johnson R. L. Stern C. D. KuehnM. Tabin C. (1995). A molecular pathway determining left-right asymmetry in chick embryogenesis. Cell 82 (5), 803–814. 10.1016/0092-8674(95)90477-8 7671308

[B25] Levin M. Pagan S. Roberts D. J. Cooke J. Kuehn M. R. Tabin C. J. (1997). Left/right patterning signals and the independent regulation of different aspects of situs in the chick embryo. Dev. Biol. 189 (1), 57–67. 10.1006/dbio.1997.8662 9281337

[B26] Lewis C. Krieg P. A. (2014). Reagents for developmental regulation of Hedgehog signaling. Methods 66 (3), 390–397. 10.1016/j.ymeth.2013.08.022 23981360

[B27] Lobikin M. Wang G. Xu J. Hsieh Y. W. Chuang C. F. Lemire J. M. (2012). Early, nonciliary role for microtubule proteins in left-right patterning is conserved across kingdoms. Proc. Natl. Acad. Sci. U. S. A. 109 (31), 12586–12591. 10.1073/pnas.1202659109 22802643 PMC3412009

[B28] Logan M. Pagan-Westphal S. M. Smith D. M. Paganessi L. Tabin C. J. (1998). The transcription factor Pitx2 mediates situs-specific morphogenesis in response to left-right asymmetric signals. Cell 94 (3), 307–317. 10.1016/s0092-8674(00)81474-9 9708733

[B29] Lombardo V. A. Heise M. Moghtadaei M. Bornhorst D. Manner J. Abdelilah-Seyfried S. (2019). Morphogenetic control of zebrafish cardiac looping by Bmp signaling. Development 146 (22), dev180091. 10.1242/dev.180091 31628109

[B30] Maerker M. Getwan M. Dowdle M. E. McSheene J. C. Gonzalez V. Pelliccia J. L. (2021). Bicc1 and Dicer regulate left-right patterning through post-transcriptional control of the Nodal inhibitor Dand5. Nat. Commun. 12 (1), 5482. 10.1038/s41467-021-25464-z 34531379 PMC8446035

[B31] Manner J. (2001). Does an equivalent of the "ventral node" exist in chick embryos? A scanning electron microscopic study. Anat. Embryol. 203 (6), 481–490. 10.1007/s004290100183 11453165

[B32] Manner J. (2009). The anatomy of cardiac looping: A step towards the understanding of the morphogenesis of several forms of congenital cardiac malformations. Clin. Anat. 22 (1), 21–35. 10.1002/ca.20652 18661581

[B33] Matsui T. Bessho Y. (2012). Left-right asymmetry in zebrafish. Cell Mol. Life Sci. 69, 3069–3077. 10.1007/s00018-012-0985-6 22527718 PMC11115138

[B34] Meinhardt A. Eberle D. Tazaki A. Ranga A. Niesche M. Wilsch-Brauninger M. (2014). 3D reconstitution of the patterned neural tube from embryonic stem cells. Stem Cell Rep. 3 (6), 987–999. 10.1016/j.stemcr.2014.09.020 PMC4264068 25454634

[B35] Motoyama J. Milenkovic L. Iwama M. Shikata Y. Scott M. P. Hui C. c. (2003). Differential requirement for Gli2 and Gli3 in ventral neural cell fate specification. Dev. Biol. 259 (1), 150–161. 10.1016/s0012-1606(03)00159-3 12812795

[B36] Narita T. Ishii Y. Nohno T. Noji S. YaSugi S. (1998). Sonic hedgehog expression in developing chicken digestive organs is regulated by epithelial-mesenchymal interactions. Dev. Growth Differ. 40 (1), 67–74. 10.1046/j.1440-169x.1998.t01-5-00008.x 9563912

[B37] Nieuwkoop P. D. Faber J. (1994). Normal table of *Xenopus laevis* (daudin). New York: Garland Publishing Inc.

[B38] Nonaka S. Yoshiba S. Watanabe D. Ikeuchi S. Goto T. Marshall W. F. (2005). De novo formation of left-right asymmetry by posterior tilt of nodal cilia. PLoS Biol. 3 (8), e268. 10.1371/journal.pbio.0030268 16035921 PMC1180513

[B39] Onjiko R. M. Morris S. E. Moody S. A. Nemes P. (2016). Single-cell mass spectrometry with multi-solvent extraction identifies metabolic differences between left and right blastomeres in the 8-cell frog (Xenopus) embryo. Analyst 141 (12), 3648–3656. 10.1039/c6an00200e 27004603 PMC4899105

[B40] Otto A. Pieper T. Viebahn C. Tsikolia N. (2014). Early left-right asymmetries during axial morphogenesis in the chick embryo. Genesis 52 (6), 614–625. 10.1002/dvg.22773 24648137

[B41] Pagan-Westphal S. M. Tabin C. J. (1998). The transfer of left-right positional information during chick embryogenesis. Cell 93 (1), 25–35. 10.1016/s0092-8674(00)81143-5 9546389

[B42] Patel K. Isaac A. Cooke J. (1999). Nodal signalling and the roles of the transcription factors SnR and Pitx2 in vertebrate left-right asymmetry. Curr. Biol. 9 (11), 609–612. 10.1016/s0960-9822(99)80267-x 10359698

[B43] Pieper T. Carpaij M. Reinermann J. Surchev L. Viebahn C. Tsikolia N. (2020). Matrix-filled microcavities in the emerging avian left-right organizer. Dev. Dyn. 249 (4), 496–508. 10.1002/dvdy.133 31729123

[B44] Rebagliati M. R. Toyama R. FriCke C. Haffter P. Dawid I. B. (1998). Zebrafish nodal-related genes are implicated in axial patterning and establishing left-right asymmetry. Dev. Biol. 199 (2), 261–272. 10.1006/dbio.1998.8935 9698446

[B45] Ribes V. Balaskas N. Sasai N. Cruz C. Dessaud E. Cayuso J. (2010). Distinct Sonic Hedgehog signaling dynamics specify floor plate and ventral neuronal progenitors in the vertebrate neural tube. Genes Dev. 24 (11), 1186–1200. 10.1101/gad.559910 20516201 PMC2878655

[B46] Roelink H. Augsburger A. Heemskerk J. Korzh V. Norlin S. Ruiz i AltAbA A. (1994). Floor plate and motor neuron induction by vhh-1, a vertebrate homolog of hedgehog expressed by the notochord. Cell 76 (4), 761–775. 10.1016/0092-8674(94)90514-2 8124714

[B47] Schroder S. S. Tsikolia N. Weizbauer A. Hue I. Viebahn C. (2016). Paraxial nodal expression reveals a novel conserved structure of the left-right organizer in four mammalian species. Cells Tissues Organs 201 (2), 77–87. 10.1159/000440951 26741372

[B48] Schweickert A. Vick P. Getwan M. Weber T. Schneider I. Eberhardt M. (2010). The nodal inhibitor Coco is a critical target of leftward flow in Xenopus. Curr. Biol. 20 (8), 738–743. 10.1016/j.cub.2010.02.061 20381352

[B49] Schweickert A. Weber T. Beyer T. Vick P. Bogusch S. Feistel K. (2007). Cilia-driven leftward flow determines laterality in Xenopus. Curr. Biol. 17 (1), 60–66. 10.1016/j.cub.2006.10.067 17208188

[B50] Stephen L. A. Johnson E. J. Davis G. M. McTeir L. Pinkham J. Jaberi N. (2014). The chicken left right organizer has nonmotile cilia which are lost in a stage-dependent manner in the talpid(3) ciliopathy. Genesis 52 (6), 600–613. 10.1002/dvg.22775 24700455 PMC4314677

[B51] Sydow H. G. Pieper T. Viebahn C. Tsikolia N. (2017). An early chick embryo culture device for extended continuous observation. Methods Mol. Biol. 1650, 309–317. 10.1007/978-1-4939-7216-6_21 28809031

[B52] Szenker-Ravi E. Ott T. Khatoo M. de Bellaing A. M. Goh W. X. Chong Y. L. (2022). Discovery of a genetic module essential for assigning left-right asymmetry in humans and ancestral vertebrates. Nat. Genet. 54 (1), 62–72. 10.1038/s41588-021-00970-4 34903892

[B53] Tisler M. Wetzel F. Mantino S. Kremnyov S. Thumberger T. Schweickert A. (2016). Cilia are required for asymmetric nodal induction in the sea urchin embryo. BMC Dev. Biol. 16 (1), 28. 10.1186/s12861-016-0128-7 27553781 PMC4994401

[B54] Todd L. Fischer A. J. (2015). Hedgehog signaling stimulates the formation of proliferating Muller glia-derived progenitor cells in the chick retina. Development 142 (15), 2610–2622. 10.1242/dev.121616 26116667 PMC4529028

[B55] Tsiairis C. D. McMahon A. P. (2009). An Hh-dependent pathway in lateral plate mesoderm enables the generation of left/right asymmetry. Curr. Biol. 19 (22), 1912–1917. 10.1016/j.cub.2009.09.057 19879143 PMC2787870

[B56] Tsikolia N. Schroder S. Schwartz P. Viebahn C. (2012). Paraxial left-sided nodal expression and the start of left-right patterning in the early chick embryo. Differentiation. 84 (5), 380–391. 10.1016/j.diff.2012.09.001 23142734

[B57] Tsikolia N. (2006). The role and limits of a gradient based explanation of morphogenesis: A theoretical consideration. Int. J. Dev. Biol. 50 (2-3), 333–340. 10.1387/ijdb.052053nt 16479501

[B58] Tsukui T. Capdevila J. Tamura K. Ruiz-Lozano P. Rodriguez-Esteban C. Yonei-Tamura S. (1999). Multiple left-right asymmetry defects in Shh(-/-) mutant mice unveil a convergence of the shh and retinoic acid pathways in the control of Lefty-1. Proc. Natl. Acad. Sci. U. S. A. 96 (20), 11376–11381. 10.1073/pnas.96.20.11376 10500184 PMC18041

[B59] Vandenberg L. N. Lemire J. M. Levin M. (2013). Serotonin has early, cilia-independent roles in Xenopus left-right patterning. Dis. Model. Mech. 6 (1), 261–268. 10.1242/dmm.010256 22899856 PMC3529356

[B60] Waddington C. H. (1932). Experiments on the development of chick and duck embryos, cultivated *in vitro* . Philosophical Trans. R. Soc. Lond. Ser. B, Contain. Pap. a Biol. Character 221, 179–230. 10.1098/rstb.1932.0003

[B61] Waddington C. H. (1936). The origin of competence for lens formation in the Amphibia. J. Exp. Biol. 13 (1), 86–91. 10.1242/jeb.13.1.86

[B62] Warner J. F. Miranda E. L. McClay D. R. (2016). Contribution of hedgehog signaling to the establishment of left-right asymmetry in the sea urchin. Dev. Biol. 411 (2), 314–324. 10.1016/j.ydbio.2016.02.008 26872875 PMC4790456

[B63] Weisheit G. Mertz D. Schilling K. Viebahn C. (2002). An efficient *in situ* hybridization protocol for multiple tissue sections and probes on miniaturized slides. Dev. Genes Evol. 212 (8), 403–406. 10.1007/s00427-002-0258-8 12203097

[B64] Zhu X. Shi C. Zhong Y. Liu X. Yan Q. Wu X. (2020). Cilia-driven asymmetric Hedgehog signalling determines the amphioxus left-right axis by controlling Dand5 expression. Development 147 (1), dev182469. 10.1242/dev.182469 31826864

