# Supplementary material for: Nodal asymmetry and hedgehog signaling during vertebrate left–right symmetry breaking
Source: Front Cell Dev Biol. 2022 Sep 12;10:957211. doi: 10.3389/fcell.2022.957211 (PMC9511907; doi:10.3389/fcell.2022.957211)
Supplement: Supplementary file 1 [file Table1.pdf]

SUPPLEMENTARY TABLE 1

A.

	treatment			
	SAG		control	
**nodal phenotype**	symmetric	asymmetric	symmetric	asymmetric
stage 5/6	20	1	0	18

B.

	treatment			
	SAG		control	
**pitx2 phenotype**	symmetric	asymmetric	symmetric	asymmetric
stage 6+/8	9	0	0	8

Left–right patterning in SAG-treated embryos and control: *nodal* (A) and *pitx2* (B) expression.
