# Supplementary material for: Nodal asymmetry and hedgehog signaling during vertebrate left–right symmetry breaking
Source: Front Cell Dev Biol. 2022 Sep 12;10:957211. doi: 10.3389/fcell.2022.957211 (PMC9511907; doi:10.3389/fcell.2022.957211)
Supplement: Supplementary file 2 [file Table2.pdf]

SUPPLEMENTARY TABLE 2

previously reported	present report
implantation of *shh* expressing cells at stage 4 (Levin et al., 1995) or beads soaked with shh protein at stage 5 (Levin et al., 1997, Logan et al., 1998) at the right side of the node	global treatment with smoothened agonist at stage 4
ectopic Shh synthesis or Shh release at the right side of the node at stage 5	non-local activation of hedgehog signaling
i. *nodal* in the right LPM at stage 9 (Levin et al., 1995)<br>ii. *pitx2* in the right LPM at stage 8 (Logan et al., 1998)<br>iii. randomisation of cardiac looping after ectopic shh treatment(Levin et al., 1997) ;<br>but both randomisation of cardiac looping and bilaterally symmetrical heart after ectopic *pitx2* expression (Logan et al., 1998)	i. ectopic nodal expression in paraxial mesoderm from stage 5+<br>ii. symmetric and premature *nodal* domain in the LPM from stage 6<br>iii. symmetric and immature *Pitx2* expression from stage 6+<br>iiii. both randomisation of cardiac looping and severe effects on heart formation
i. ectopic Shh is sufficient to induce *nodal* (Levin et al., 1995)<br>ii. induction of *pitx2* is mediates by Nodal (Logan et al., 1998)<br>iii. Shh promotes heart looping while Pitx2 isomeric heart with mirrored left identity (Logan et al., 1998)	i. elevated global hedgehog activity induces *nodal* in specific competent domains<br>ii. randomisation of heart looping is affected by nodal-pitx2 mediated mechanisms<br>iii. severe cardiac malformations are caused by hedgehog-mediated failure of intestinal portal development
	i. investigation of competence<br>ii. investigation of hedgehog effects on heart development

Hedgehog signaling in the chick left–right patterning: compilation of previous results concerning the role of hedgehog signaling in left–right patterning in comparison to the present report.
