# Supplementary material for: Nodal asymmetry and hedgehog signaling during vertebrate left–right symmetry breaking
Source: Front Cell Dev Biol. 2022 Sep 12;10:957211. doi: 10.3389/fcell.2022.957211 (PMC9511907; doi:10.3389/fcell.2022.957211)
Supplement: Supplementary file 3 [file Image2.pdf]

SUPPLEMENTARY FIGURE S2

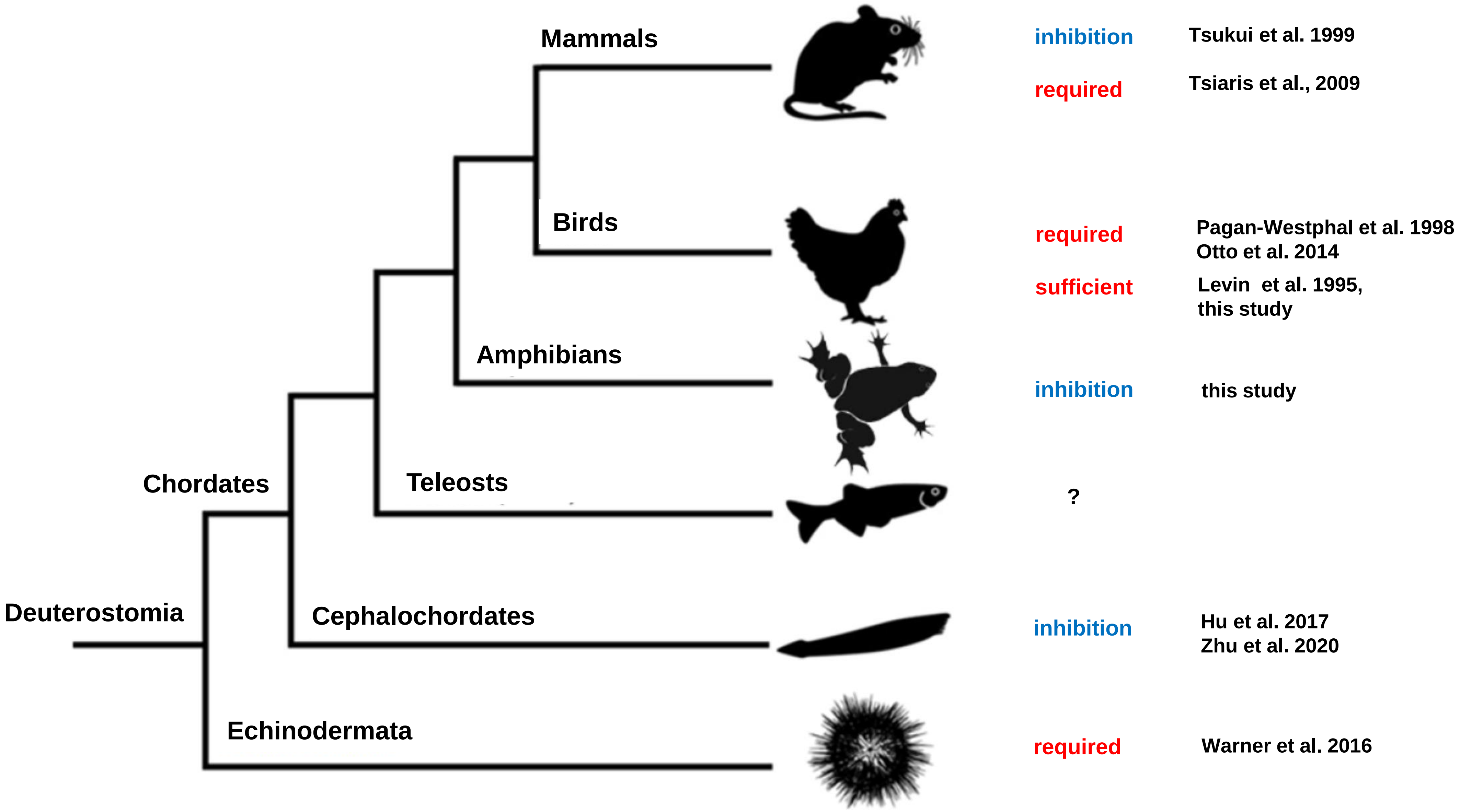

Hedgehog signaling and left–right patterning in Deuterostomia. The red font is used for observed stimulating effect which is subdivided into “sufficient” and “required” (necessary) for *nodal* expression while the blue font is used for reported inhibition of *nodal* domain.
