# Supplementary material for: Nodal asymmetry and hedgehog signaling during vertebrate left–right symmetry breaking
Source: Front Cell Dev Biol. 2022 Sep 12;10:957211. doi: 10.3389/fcell.2022.957211 (PMC9511907; doi:10.3389/fcell.2022.957211)
Supplement: Supplementary file 4 [file DataSheet1.PDF]

Supplementary methods

Embryos

All protocols were developed in accordance with European convention for the protection of vertebral animals used for experimental and other scientific purposes (Strasbourg,1986) and approved by Ethic Committee for animal research of the Koltzov Institute of Developmental biology (approval number 58). Animals were anesthetized with MS222 for *in vitro* fertilization and tadpoles were anesthetized with MS222 prior to fixation.

Cloning and antisense RNA probes

Digoxigenin-labeled mRNA probes for *nodal* and *pitx2* were produced using the previously published plasmid DNAs (Levin et al., 1995; Logan et al., 1998) and protocols (Weisheit et al., 2002).
nodal1 cDNA was cloned using following primers:
*nodal1*_forward: TTAATGCAAACCCTCCTTCTACCA
*nodal1*_reverse: TCAAAACAACCTCATCTCCCTCAT
Amplified fragments were cloned into the pAL2 vector (Evrogen, Russia). PCR products from the plasmids were amplified with T7 and Sp6 oligos and used for generation of digoxigenin-labeled RNA (Roche) antisense probes.

In situ RNA hybridization

Rehydrated chicken embryos transferred to nylon baskets were treated with 10 mg/ml proteinase K (Roche, Grenzach-Wyhlen, Germany) in PBT for 10 min, postfixed in 0.2% glutaraldehyde/PBT for 20 min. Baskets with embryonic disk were then transferred to sterile screw-top PVC tubes (Bibby Sterilin, Staffordshire, UK) for 1 h prehybridization at 70 °C in a heating block in hybridization buffer [50% formamide, 1.4X SSC, 0.1% 0.5 mM EDTA, 50 µg/ml t-RNA, 0.2% Tween-20, 0.5% CHAPS, 50 µg/ml heparin (AppliChem, Darmstadt, Germany)]. The embryos were hybridized overnight at 70 °C in a hybridization buffer with 1 µg/ml digoxygenin-labeled cRNA denatured at 95 °C. In the next step embryos were washed in prewarmed hybridization buffer and MABT (100 mM maleic acid, 150 mM NaCl, 0.1% Tween-20, pH 7.5). Thereafter, the baskets with embryos were transferred in MABT with 2% Roche blocking reagent and 20% heat-inactivated goat serum. Hybridized RNA was visualized with antidigoxigenin antibody coupled to alkaline phosphatase and BM purple substrate (both Roche, Mannheim, Germany). To initiate the color reaction, embryos were transferred to Petri dishes filled with the substrate, and the reaction was allowed to proceed at room temperature in the dark for 2–5 days.

Wild-type or SAG-treated *Xenopus laevis* embryos were fixed at the stage 24 in 4% formaldehyde in PBS for 2 hours, stored in 100 % ethanol at -20 °C. Whole-mount embryos were processed following modified published protocol (Harland, 1991). All steps were performed in glass vials. Briefly: fixed samples were rehydrated in 75% ethanol, 50% ethanol and 25% ethanol in PBS with 0.1% Tween-20 (PTw) for 5 minutes each, washed three times in PTw for 5 minutes each, then incubated in 10 µg/ml proteinase K in PTw for 12 minutes. Digestion was stopped by washing twice in freshly prepared

triethanolamine solution (TEA), then in 0.25% and 0.5% acetic anhydride in TEA for 5 minutes each. Samples were washed twice in PTw for 5 minutes each and re-fixed in 4% paraformaldehyde in PTw for 30 minutes at room temperature. Samples were washed four times in PTw for 5 minutes each, in 20% hybridization buffer (HB) in PTw and 100% HB for 10 minutes each and prehybridized in HB at 60°C for 12 h. Samples were hybridized at 60°C overnight in probe solution (HB containing 300 ng/ml of antisense RNA probe). Samples were washed in prehybridization solution for 1 hour at 60°C, then three times in 2x SSC (pH 7) at 60°C for 20 minutes each, twice in 0.2x SSC (pH 7.0) at 60°C for 30 minutes each and finally twice in maleic acid buffer (MAB; 0.1M maleic acid, 0.15M NaCl (pH 7.5)) at room temperature for 15 minutes each. Samples were treated with blocking solution (1% blocking reagent Roche/MAB) for 2,5 h at room temperature and then with 0.1% anti-digoxigenin AP antibodies in blocking solution overnight at 4°C. Samples were washed ten times for 1 h in MAB at room temperature and stained in BM purple substrate (Boehringer) at 4°C in the dark (a few hours to several days). After staining, samples were fixed in 4% formaldehyde/PBS for 2 hours, bleached in a solution of 0.5x SSC, 5% formamide and 5% H_2O_2 for 1 to 4 h under bright light to remove pigmentation, re-fixed in 4% formaldehyde/PBS for 2 hours, transferred to ethanol and then to glycerine for long-term storage at -20°C. Samples were examined at Olympus SZX9 stereomicroscope (Olympus, Japan) and embedded in Technovit 8100® (Heraeus Kulzer, Wehrheim, Germany) according to the manufacturers' protocol. 5 µm sections were cut at sectional planes predefined in whole-mount views and photographed using bright-field illumination or Nomarski contrast.

Data analysis

Statistical significance was determined using the R program (R Development Core Team, 2004).

To analyze *nodal1* expression in stage 24 embryos we divided the observed phenotypes into groups and counted the number of samples in each group. Statistical calculations of gene expression patterns of SAG-modulated embryos were performed with two-proportions Z-test.

References

Harland, R.M., 1991. In situ hybridization: an improved whole-mount method for Xenopus embryos. Methods Cell Biol 36, 685-695.

Levin, M., Johnson, R.L., Stern, C.D., Kuehn, M., Tabin, C., 1995. A molecular pathway determining left-right asymmetry in chick embryogenesis. Cell 82, 803-814.

Logan, M., Pagan-Westphal, S.M., Smith, D.M., Paganessi, L., Tabin, C.J., 1998. The transcription factor Pitx2 mediates situs-specific morphogenesis in response to left-right asymmetric signals. Cell 94, 307-317.

Weisheit, G., Mertz, D., Schilling, K., Viebahn, C., 2002. An efficient in situ hybridization protocol for multiple tissue sections and probes on miniaturized slides. Dev Genes Evol 212, 403-406.
