# Supplementary material for: Nodal asymmetry and hedgehog signaling during vertebrate left–right symmetry breaking
Source: Front Cell Dev Biol. 2022 Sep 12;10:957211. doi: 10.3389/fcell.2022.957211 (PMC9511907; doi:10.3389/fcell.2022.957211)
Supplement: Supplementary file 5 [file Image1.pdf]

SUPPLEMENTARY FIGURE S1

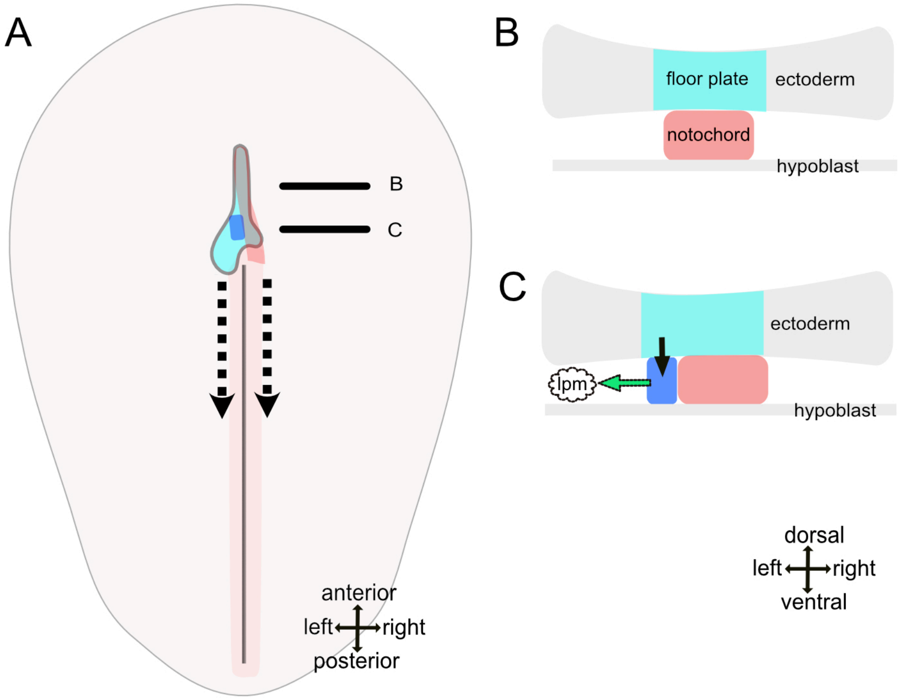

Proposed mechanism of molecular left–right patterning as a consequence of asymmetric node morphogenesis in the chick. A: schematic dorsal view of chick embryo at early stage 5 after node rotation. B,C: transversal sections at levels shown in (A). The notochord is marked in red, the floor plate is labeled in turquoise and blue indicates the paraxial *nodal* domain. Dashed arrows in (A) indicate a shift of the node during notochord elongation and concomitant streak regression, black arrow in (C) indicates the proposed induction of the paraxial *nodal* expression by shh from the notochord, green arrow in (C) indicates the proposed subsequent induction of *nodal* expression in the prospective lateral plate mesoderm (lpm) by nodal ligand from the paraxial domain. A is modified version of Figure 7B published in (Kremnyov et al. 2018).
